# Crystal structure of 1-(2-amino­phen­yl)-3-phenyl­urea

**DOI:** 10.1107/S2056989014028175

**Published:** 2015-01-10

**Authors:** Joel T. Mague, Shaaban K. Mohamed, Mehmet Akkurt, Omran A. Omran, Mustafa R. Albayati

**Affiliations:** a Department of Chemistry, Tulane University, New Orleans, LA 70118, USA; b Chemistry and Environmental Division, Manchester Metropolitan University, Manchester M1 5GD, England; c Chemistry Department, Faculty of Science, Minia University, 61519 El-Minia, Egypt; d Department of Physics, Faculty of Sciences, Erciyes University, 38039 Kayseri, Turkey; e Chemistry Department, Faculty of Science, Sohag University, 82524 Sohag, Egypt; f Kirkuk University, College of Science, Department of Chemistry, Kirkuk, Iraq

**Keywords:** crystal structure, urea derivatives, N—H⋯N hydrogen bonds, N—H⋯O hydrogen bonds, twinned structure

## Abstract

In the title compound, C_13_H_13_N_3_O, the phenyl ring makes a dihedral angle of 47.0 (1)° with the mean plane of the –NC(=O)N– unit, while the dihedral angle between the latter mean plane and the amino­phenyl ring is 84.43 (7)°. In the crystal, mol­ecules are linked *via* N—H⋯O hydrogen bonds involving the central –NHC(=O)NH– units, forming chains running parallel to the *b* axis. These chains associate with one another *via* N—H⋯O and N—H⋯N hydrogen bonds, from the pendant amino groups to the –NHC(=O)NH– units of adjacent mol­ecules, forming columns propagating along [010]. The structure was refined as a two-component twin with a 0.933 (3):0.067 (3) domain ratio.

## Related literature   

For industrial applications of urea-containing compounds, see: Kapuscinska & Nowak (2014 ▸); Doyle & Jacobsen (2007 ▸); Helm *et al.* (1989 ▸). For the wide spectrum of biological activities of urea scaffold compounds, see: Upadhayaya *et al.* (2009 ▸); Khan *et al.* (2008 ▸), Seth *et al.* (2004 ▸); Kaymakçıoğlu *et al.* (2005 ▸); Yip & Yang (1986 ▸). For details of the use of the TWINROTMAT routine in *PLATON*, see: Spek (2009 ▸).
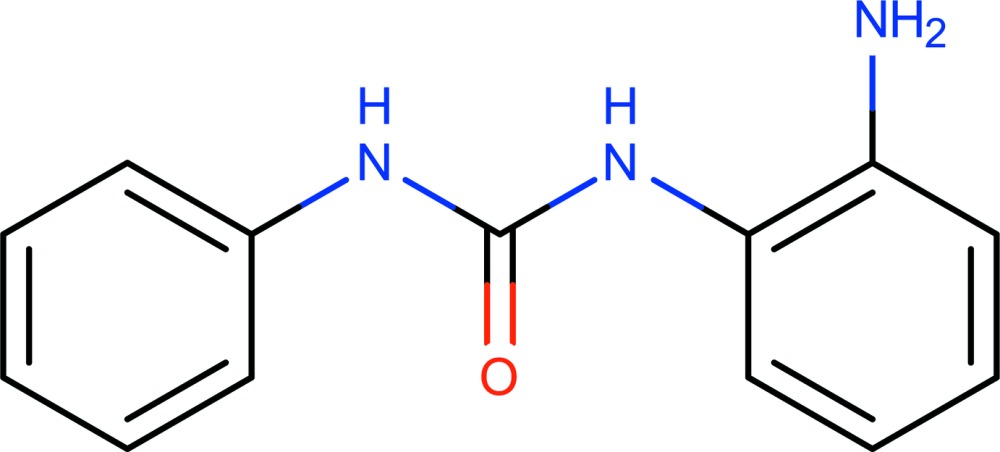


## Experimental   

### Crystal data  

C_13_H_13_N_3_O
*M* *_r_* = 227.26
Monoclinic, 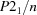

*a* = 16.1742 (4) Å
*b* = 4.5667 (1) Å
*c* = 16.3259 (4) Å
β = 106.548 (1)°
*V* = 1155.93 (5) Å^3^
*Z* = 4
Cu *K*α radiation
μ = 0.69 mm^−1^
*T* = 150 K
0.20 × 0.12 × 0.09 mm

### Data collection  

Bruker D8 VENTURE PHOTON 100 CMOS diffractometer
Absorption correction: multi-scan (*SADABS*; Bruker, 2014 ▸) *T*_min_ = 0.89, *T*_max_ = 0.94
21843 measured reflections
2282 independent reflections
2084 reflections with *I* > 2σ(*I*)
*R*_int_ = 0.035

### Refinement  

*R*[*F* ^2^ > 2σ(*F* ^2^)] = 0.049
*wR*(*F* ^2^) = 0.136
*S* = 1.11
2282 reflections
155 parameters
H-atom parameters constrained
Δρ_max_ = 0.30 e Å^−3^
Δρ_min_ = −0.22 e Å^−3^

### 

Data collection: *APEX2* (Bruker, 2014 ▸); cell refinement: *SAINT* (Bruker, 2014 ▸); data reduction: *SAINT*; program(s) used to solve structure: *SHELXT* (Sheldrick, 2008 ▸); program(s) used to refine structure: *SHELXL2014* (Sheldrick, 2008 ▸); molecular graphics: *DIAMOND* (Brandenburg & Putz, 2012 ▸); software used to prepare material for publication: *SHELXTL* (Sheldrick, 2008 ▸) and *PLATON* (Spek, 2009 ▸).

## Supplementary Material

Crystal structure: contains datablock(s) global, I. DOI: 10.1107/S2056989014028175/su5051sup1.cif

Structure factors: contains datablock(s) I. DOI: 10.1107/S2056989014028175/su5051Isup2.hkl

Supporting information file. DOI: 10.1107/S2056989014028175/su5051Isup3.cml

**Fig. 1.**
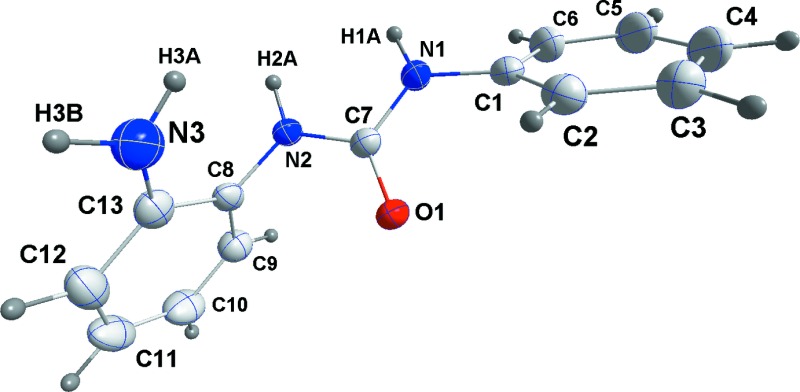
The molecular structure of the title compound, with atom labelling. Displacement ellipsoids are drawn at the 50% probability level.

**Fig. 2.**
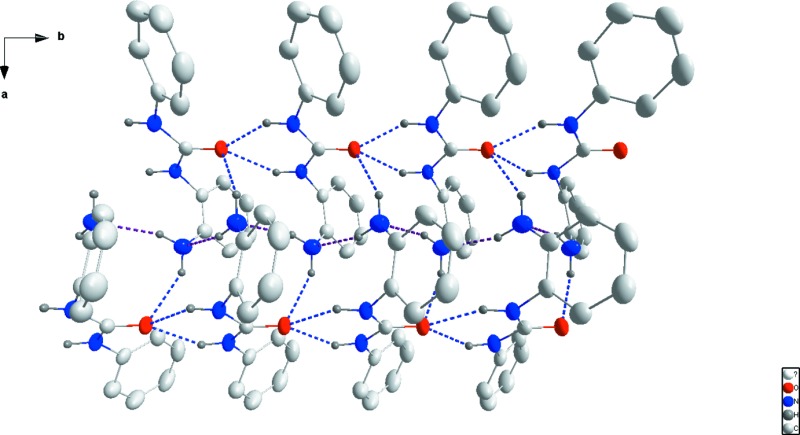
A view along the c axis of the crystal packing of the title compound. The N—H···O and N—H···N hydrogen bonds are shown by blue and violet dashed lines, respectively (see Table 1 for details).

. DOI: 10.1107/S2056989014028175/su5051fig1.tif
The mol­ecular structure of the title compound, with atom labelling. Displacement ellipsoids are drawn at the 50% probability level.

c . DOI: 10.1107/S2056989014028175/su5051fig2.tif
A view along the *c* axis of the crystal packing of the title compound. The N—H⋯O and N—H⋯N hydrogen bonds are shown by blue and violet dashed lines, respectively (see Table 1 for details).

CCDC reference: 1041048

Additional supporting information:  crystallographic information; 3D view; checkCIF report

## Figures and Tables

**Table 1:** Hydrogen-bond geometry (, )

*D*H*A*	*D*H	H*A*	*D* *A*	*D*H*A*
N2H2*A*O1^i^	0.91	2.13	2.932(2)	147
N1H1*A*O1^i^	0.91	1.94	2.771(2)	151
N3H3*A*N3^ii^	0.91	2.19	3.057(3)	160
N3H3*B*O1^ii^	0.91	2.24	3.004(2)	141

Symmetry codes: (i) 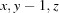; (ii) 
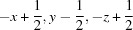
.

## supplementary crystallographic information

### S1. Comment

Compounds bearing a urea linkage have attracted the interest of many researchers due to the variety of their applications in both of medicinal and industrial fields. One of the most important class of compounds that are used in the cosmetic industry are urea-containing compounds due to their effective moisturizing properties (Kapuscinska & Nowak, 2014). Urea-linked glycosides serve as small-molecule H-bond donors in asymmetric catalysis (Doyle & Jacobsen, 2007), and are currently employed in the forestry product industry, for example as adhesive mixtures to reduce the level of toxic phenol in furniture and building materials (Helm *et al.*, 1989). Some urea derivatives possess valuable antituberculosis, antibacterial and anticonvulsant properties (Upadhayaya *et al.*, 2009; Khan *et al.*, 2008, Sett *et al.*, 2004; Koçyiğit-Kaymakçıoğlu *et al.*, 2005). Compounds such as Thidiazuron have mimicked the effect of benzyladenine (BA) in the Ca^2+^ and cytokinin systems (Yip *et al.*, 1986). Based on such findings we report in this study the synthesis and crystal structure of the title compound.

The phenyl ring makes a dihedral angle of 47.0 (1)° with the mean plane of atoms N1/N2/C7/O1 while the dihedral angle between the latter unit and the aminophenyl ring is 84.43 (7)°.

In the crystal, N1—H1A···O1^i^ and N2—H2a···O1^i^ hydrogen bonds link chains of molecules running parallel to the *b* axis (Fig. 2 and Table 1). Pairs of chains are further associated through N3—H3A···N3^ii^ and N3—H3B···O1^ii^ hydrogen bonds (Table 1 and Fig. 2), forming columns propagating along [010].

### S2. Experimental

A mixture of 0.01 mol (2.06 g m) of *N*-phenylmorpholine-4-carboxamide and 0.01 mol (1.08 g m) benzene-1,2-diamine in 20 ml of ethanol was heated under reflux for 10 h. On cooling, the resulting solid product was collected by filtration, washed with a little cold ethanol and dried under vacuum. Colourless crystals suitable for X-ray diffraction were obtained by recrystallization of the product from ethanol (m.p.: 495 K; yield: 73%).

### S3. Refinement

The C-bound H atoms were placed in calculated positions (C—H = 0.95 Å) while those attached to nitrogen were placed in locations derived from a difference Fourier map and their parameters adjusted to give N—H = 0.91 Å. They were all treated as riding atoms with U_iso_(H) = 1.2U_eq_(N,C). In the final stages of the refinement, analysis of the data with the *TWINROTMAT* routine in *PLATON* (Spek, 2009) indicated the presence of a minor twin component rotated by approximately 180° about [101] and the data were finally refined as a 2-component twin (BASF = 0.067).

### Crystal data

**Table:**

C_13_H_13_N_3_O	*F*(000) = 480
*M**_r_* = 227.26	*D*_x_ = 1.306 Mg m^−^^3^
Monoclinic, *P*2_1_/*n*	Cu *K*α radiation, λ = 1.54178 Å
*a* = 16.1742 (4) Å	Cell parameters from 9955 reflections
*b* = 4.5667 (1) Å	θ = 3.4–72.4°
*c* = 16.3259 (4) Å	µ = 0.69 mm^−^^1^
β = 106.548 (1)°	*T* = 150 K
*V* = 1155.93 (5) Å^3^	Column, colourless
*Z* = 4	0.20 × 0.12 × 0.09 mm

### Data collection

**Table:**

Bruker D8 VENTURE PHOTON 100 CMOS diffractometer	2282 independent reflections
Radiation source: INCOATEC IµS micro–focus source	2084 reflections with *I* > 2σ(*I*)
Mirror monochromator	*R*_int_ = 0.035
Detector resolution: 10.4167 pixels mm^-1^	θ_max_ = 72.5°, θ_min_ = 2.8°
ω scans	*h* = −19→17
Absorption correction: multi-scan (*SADABS*; Bruker, 2014)	*k* = −5→5
*T*_min_ = 0.89, *T*_max_ = 0.94	*l* = −20→20
21843 measured reflections	

### Refinement

**Table:**

Refinement on *F*^2^	Primary atom site location: structure-invariant direct methods
Least-squares matrix: full	Secondary atom site location: difference Fourier map
*R*[*F*^2^ > 2σ(*F*^2^)] = 0.049	Hydrogen site location: mixed
*wR*(*F*^2^) = 0.136	H-atom parameters constrained
*S* = 1.11	*w* = 1/[σ^2^(*F*_o_^2^) + (0.054*P*)^2^ + 0.8485*P*] where *P* = (*F*_o_^2^ + 2*F*_c_^2^)/3
2282 reflections	(Δ/σ)_max_ < 0.001
155 parameters	Δρ_max_ = 0.30 e Å^−^^3^
0 restraints	Δρ_min_ = −0.22 e Å^−^^3^

### Special details

**Table:**

Geometry. All e.s.d.'s (except the e.s.d. in the dihedral angle between two l.s. planes)are estimated using the full covariance matrix. The cell e.s.d.'s are takeninto account individually in the estimation of e.s.d.'s in distances, anglesand torsion angles; correlations between e.s.d.'s in cell parameters are onlyused when they are defined by crystal symmetry. An approximate (isotropic)treatment of cell e.s.d.'s is used for estimating e.s.d.'s involving l.s. planes.
Refinement. Refinement of *F*^2^ against ALL reflections. The weighted *R*-factor *wR* and goodness of fit *S* are based on *F*^2^, conventional *R*-factors *R* are based on *F*, with *F* set to zero for negative *F*^2^. The threshold expression of *F*^2^ > σ(*F*^2^) is used only for calculating *R*-factors(gt) *etc*. and is not relevant to the choice of reflections for refinement. *R*-factors based on *F*^2^ are statistically about twice as large as those based on *F*, and *R*- factors based on ALL data will be even larger. H-atoms attached to carbon were placed in calculated positions (C—H = 0.95 Å) while those attached to nitrogen were placed in locations derived from a difference map and their parameters adjusted to give N—H = 0.91 Å. All were included as riding contributions with isotropic displacement parameters 1.2 times those of the attached atoms. In the final stages of the refinement, analysis of the data with the *TWINROTMAT* routine in *PLATON* (Spek, 2014) indicated the presence of a minor twin component rotated by approximately 180° about *b* and the data were finally refined as a 2-component twin.

### Fractional atomic coordinates and isotropic or equivalent isotropic displacement parameters (Å^2^)

**Table:**

	*x*	*y*	*z*	*U*_iso_*/*U*_eq_	
O1	0.44188 (9)	0.4790 (3)	0.32769 (9)	0.0300 (3)	
N1	0.48770 (11)	0.0380 (4)	0.29093 (11)	0.0309 (4)	
H1A	0.4911	−0.1584	0.3007	0.037*	
N2	0.40020 (11)	0.0618 (3)	0.38000 (10)	0.0277 (4)	
H2A	0.3974	−0.1368	0.3754	0.033*	
N3	0.22413 (12)	0.1572 (4)	0.29992 (12)	0.0413 (5)	
H3A	0.2521	0.0112	0.2807	0.050*	
H3B	0.1655	0.1521	0.2808	0.050*	
C1	0.53488 (14)	0.1492 (4)	0.23655 (13)	0.0305 (4)	
C2	0.49906 (16)	0.3542 (5)	0.17311 (13)	0.0383 (5)	
H2	0.4428	0.4293	0.1667	0.046*	
C3	0.5467 (2)	0.4474 (6)	0.11924 (16)	0.0510 (7)	
H3	0.5235	0.5916	0.0770	0.061*	
C4	0.6271 (2)	0.3331 (6)	0.12642 (18)	0.0551 (7)	
H4	0.6587	0.3957	0.0886	0.066*	
C5	0.66188 (18)	0.1280 (6)	0.18846 (17)	0.0499 (6)	
H5	0.7171	0.0472	0.1929	0.060*	
C6	0.61656 (15)	0.0386 (5)	0.24462 (15)	0.0396 (5)	
H6	0.6415	−0.0982	0.2885	0.048*	
C7	0.44317 (12)	0.2076 (4)	0.33236 (11)	0.0255 (4)	
C8	0.34822 (13)	0.2198 (4)	0.42286 (12)	0.0269 (4)	
C9	0.38565 (15)	0.3407 (5)	0.50231 (13)	0.0371 (5)	
H9	0.4453	0.3114	0.5294	0.045*	
C10	0.33681 (18)	0.5047 (6)	0.54293 (15)	0.0464 (6)	
H10	0.3628	0.5887	0.5974	0.056*	
C11	0.25015 (18)	0.5444 (5)	0.50347 (16)	0.0464 (6)	
H11	0.2164	0.6563	0.5310	0.056*	
C12	0.21204 (15)	0.4233 (5)	0.42441 (15)	0.0412 (5)	
H12	0.1521	0.4522	0.3982	0.049*	
C13	0.26029 (13)	0.2584 (5)	0.38210 (13)	0.0317 (4)	

### Atomic displacement parameters (Å^2^)

**Table:**

	*U*^11^	*U*^22^	*U*^33^	*U*^12^	*U*^13^	*U*^23^
O1	0.0377 (8)	0.0201 (7)	0.0356 (7)	−0.0009 (6)	0.0158 (6)	0.0009 (5)
N1	0.0379 (9)	0.0210 (8)	0.0393 (9)	0.0002 (7)	0.0199 (8)	0.0012 (7)
N2	0.0328 (9)	0.0195 (7)	0.0337 (8)	−0.0002 (6)	0.0139 (7)	0.0009 (7)
N3	0.0359 (10)	0.0442 (11)	0.0404 (10)	0.0007 (8)	0.0052 (8)	−0.0010 (9)
C1	0.0385 (11)	0.0244 (9)	0.0318 (10)	−0.0071 (8)	0.0154 (9)	−0.0064 (8)
C2	0.0501 (13)	0.0326 (11)	0.0342 (11)	0.0000 (10)	0.0153 (10)	−0.0020 (9)
C3	0.083 (2)	0.0362 (12)	0.0401 (12)	−0.0022 (13)	0.0283 (13)	0.0036 (10)
C4	0.0785 (19)	0.0447 (14)	0.0600 (16)	−0.0137 (13)	0.0486 (15)	−0.0063 (12)
C5	0.0494 (14)	0.0487 (14)	0.0619 (16)	−0.0061 (12)	0.0327 (13)	−0.0065 (12)
C6	0.0407 (12)	0.0385 (12)	0.0431 (12)	−0.0007 (10)	0.0174 (10)	−0.0010 (10)
C7	0.0259 (9)	0.0230 (9)	0.0269 (9)	−0.0003 (7)	0.0065 (7)	0.0004 (7)
C8	0.0330 (10)	0.0206 (9)	0.0297 (9)	0.0011 (8)	0.0131 (8)	0.0035 (7)
C9	0.0405 (12)	0.0368 (11)	0.0336 (11)	−0.0022 (9)	0.0099 (9)	−0.0012 (9)
C10	0.0632 (16)	0.0434 (13)	0.0363 (11)	−0.0043 (12)	0.0199 (11)	−0.0097 (10)
C11	0.0632 (16)	0.0364 (12)	0.0520 (14)	0.0065 (11)	0.0361 (13)	−0.0019 (11)
C12	0.0373 (12)	0.0406 (13)	0.0505 (13)	0.0083 (10)	0.0201 (10)	0.0076 (10)
C13	0.0346 (11)	0.0300 (10)	0.0322 (10)	−0.0003 (8)	0.0120 (8)	0.0053 (8)

### Geometric parameters (Å, º)

**Table:**

O1—C7	1.241 (2)	C4—C5	1.377 (4)
N1—C7	1.362 (2)	C4—H4	0.9500
N1—C1	1.419 (2)	C5—C6	1.388 (3)
N1—H1A	0.9099	C5—H5	0.9500
N2—C7	1.356 (2)	C6—H6	0.9500
N2—C8	1.433 (2)	C8—C9	1.381 (3)
N2—H2A	0.9099	C8—C13	1.399 (3)
N3—C13	1.381 (3)	C9—C10	1.387 (3)
N3—H3A	0.9101	C9—H9	0.9500
N3—H3B	0.9101	C10—C11	1.378 (4)
C1—C6	1.385 (3)	C10—H10	0.9500
C1—C2	1.394 (3)	C11—C12	1.378 (4)
C2—C3	1.391 (3)	C11—H11	0.9500
C2—H2	0.9500	C12—C13	1.400 (3)
C3—C4	1.375 (4)	C12—H12	0.9500
C3—H3	0.9500		
			
C7—N1—C1	124.19 (16)	C1—C6—C5	119.9 (2)
C7—N1—H1A	119.2	C1—C6—H6	120.0
C1—N1—H1A	116.5	C5—C6—H6	120.0
C7—N2—C8	120.03 (15)	O1—C7—N2	121.53 (17)
C7—N2—H2A	117.6	O1—C7—N1	122.64 (17)
C8—N2—H2A	121.4	N2—C7—N1	115.82 (16)
C13—N3—H3A	117.7	C9—C8—C13	120.72 (18)
C13—N3—H3B	117.0	C9—C8—N2	119.93 (18)
H3A—N3—H3B	115.7	C13—C8—N2	119.31 (17)
C6—C1—C2	119.93 (19)	C8—C9—C10	120.5 (2)
C6—C1—N1	118.56 (19)	C8—C9—H9	119.7
C2—C1—N1	121.43 (19)	C10—C9—H9	119.7
C3—C2—C1	119.2 (2)	C11—C10—C9	119.3 (2)
C3—C2—H2	120.4	C11—C10—H10	120.3
C1—C2—H2	120.4	C9—C10—H10	120.3
C4—C3—C2	120.7 (2)	C12—C11—C10	120.6 (2)
C4—C3—H3	119.6	C12—C11—H11	119.7
C2—C3—H3	119.6	C10—C11—H11	119.7
C3—C4—C5	119.9 (2)	C11—C12—C13	121.0 (2)
C3—C4—H4	120.0	C11—C12—H12	119.5
C5—C4—H4	120.0	C13—C12—H12	119.5
C4—C5—C6	120.3 (2)	N3—C13—C8	120.78 (18)
C4—C5—H5	119.9	N3—C13—C12	121.2 (2)
C6—C5—H5	119.9	C8—C13—C12	117.83 (19)
			
C7—N1—C1—C6	135.3 (2)	C7—N2—C8—C9	−84.9 (2)
C7—N1—C1—C2	−48.0 (3)	C7—N2—C8—C13	92.9 (2)
C6—C1—C2—C3	−0.8 (3)	C13—C8—C9—C10	−0.5 (3)
N1—C1—C2—C3	−177.4 (2)	N2—C8—C9—C10	177.3 (2)
C1—C2—C3—C4	2.0 (4)	C8—C9—C10—C11	0.5 (4)
C2—C3—C4—C5	−1.2 (4)	C9—C10—C11—C12	−0.1 (4)
C3—C4—C5—C6	−0.9 (4)	C10—C11—C12—C13	−0.3 (4)
C2—C1—C6—C5	−1.2 (3)	C9—C8—C13—N3	174.9 (2)
N1—C1—C6—C5	175.5 (2)	N2—C8—C13—N3	−2.9 (3)
C4—C5—C6—C1	2.1 (4)	C9—C8—C13—C12	0.1 (3)
C8—N2—C7—O1	3.2 (3)	N2—C8—C13—C12	−177.65 (17)
C8—N2—C7—N1	−177.12 (17)	C11—C12—C13—N3	−174.5 (2)
C1—N1—C7—O1	−1.9 (3)	C11—C12—C13—C8	0.2 (3)
C1—N1—C7—N2	178.46 (18)		

### Hydrogen-bond geometry (Å, º)

**Table:**

*D*—H···*A*	*D*—H	H···*A*	*D*···*A*	*D*—H···*A*
N2—H2*A*···O1^i^	0.91	2.13	2.932 (2)	147
N1—H1*A*···O1^i^	0.91	1.94	2.771 (2)	151
N3—H3*A*···N3^ii^	0.91	2.19	3.057 (3)	160
N3—H3*B*···O1^ii^	0.91	2.24	3.004 (2)	141

Symmetry codes: (i) *x*, *y*−1, *z*; (ii) −*x*+1/2, *y*−1/2, −*z*+1/2.
