# Supplementary figures and images for: Crystal structure of 1-(2-amino­phen­yl)-3-phenyl­urea
Source: Acta Crystallogr E Crystallogr Commun. 2015 Jan 10;71(Pt 2):o88–9. doi: 10.1107/S2056989014028175 (PMC4384537; doi:10.1107/S2056989014028175)

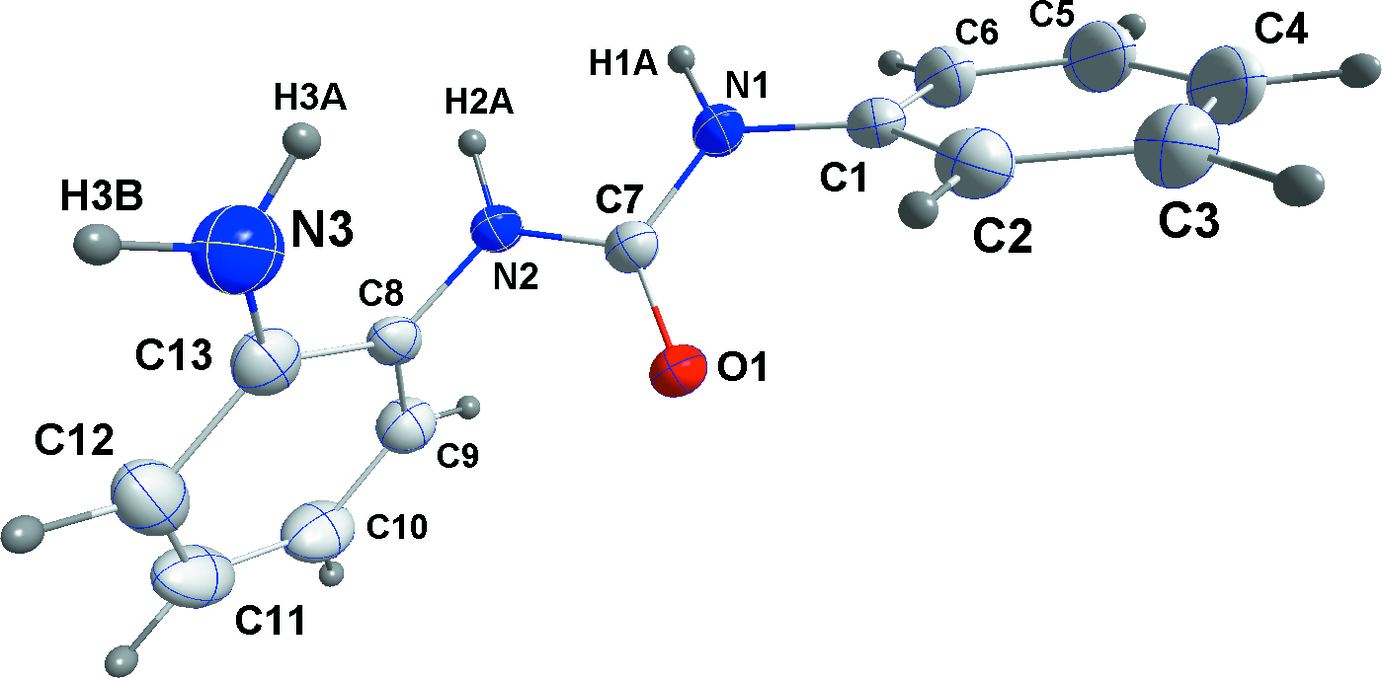

Supplement: Supplementary file 4 [file e-71-00o88-fig1.tif]

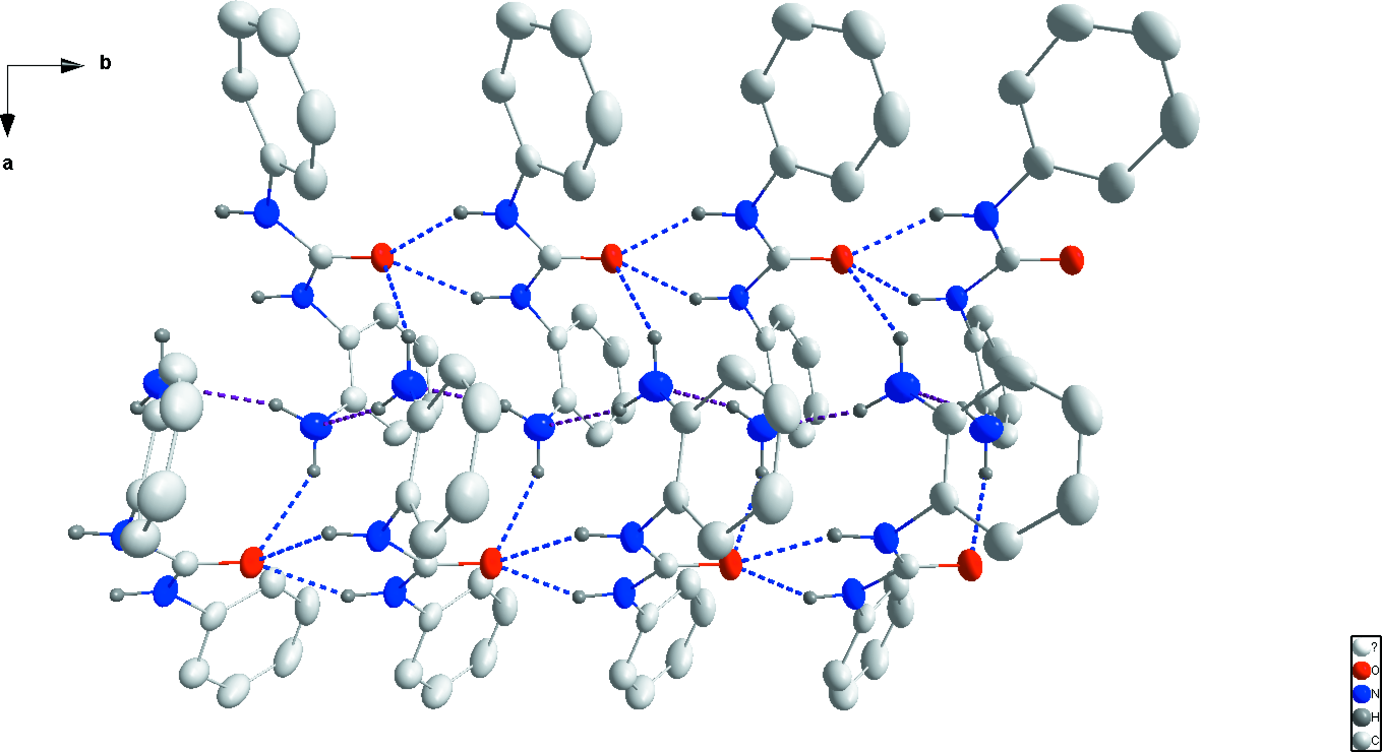

Supplement: Supplementary file 5 [file e-71-00o88-fig2.tif]
